# GPR37 promotes the malignancy of lung adenocarcinoma via TGF-β/Smad pathway

**DOI:** 10.1515/med-2021-0011

**Published:** 2020-12-05

**Authors:** Jian Wang, Min Xu, Dan-Dan Li, Wujikenayi Abudukelimu, Xiu-Hong Zhou

**Affiliations:** Department of Respiration, Midong Branch of People’s Hospital of Xinjiang Autonomous Region, 1302-17 Midong South Road, Urumqi, Xinjiang, People's Republic of China; Department of Medical, Midong Branch of People’s Hospital of Xinjiang Autonomous Region, Urumqi, Xinjiang, People's Republic of China; Department of Endocrinology, Midong Branch of People’s Hospital of Xinjiang Autonomous Region, Xinjiang, Urumqi, People's Republic of China

**Keywords:** lung adenocarcinoma, GPR37, TGF-β/Smad pathway

## Abstract

This paper aimed to research the function and in-depth mechanism of GPR37 in lung adenocarcinoma (LUAD). Herein, based on TCGA and Oncomine databases, we revealed that GPR37 was expressed at high levels in LUAD, and upregulation of GPR37 was related to the poor outcomes. Furthermore, biological function experiments *in vitro* were utilized to assess whether GPR37 impacts malignant phenotype of LUAD cells. Gain- or loss-of-function assays indicated that the upregulation of GPR37 contributed to improving the proliferation, migration, and invasion of LUAD cells *in vitro*, while knockdown of GPR37 can inhibit the malignant biological behaviors. Then, we found that depletion of GPR37 resulted in a decrease in the expression of TGF-β1 as well as the extents of Smad2 and Smad3 phosphorylation, while overexpression of GPR37 presented opposite outcomes. Altogether, our findings indicated that GPR37 is a potential oncogene of LUAD, and its promoting effects on the malignant progression of LUAD may be realized via TGF-β/Smad pathway.

## 1 Introduction

Lung cancer is a malignant tumor with the highest incidence worldwide [1]. Recently, lung adenocarcinoma (LUAD) has become a common pathological type of lung cancer, accounting for 40–50% of all lung cancers [2]. Due to the concealment of early symptoms, most of the patients (about 57%) with LUAD were diagnosed as advanced [3]. With the continuous renewal of lung cancer treatment concept, targeted drugs are considered to be effective in prolonging the survival time of patients with non-small cell lung cancer [4]. Unfortunately, the 5-year survival rate of patients with advanced LUAD is only 10–15% [1,5]. The main reason may be the drug resistance of the tumor [6]. Hence, it is necessary to search for the potential biomarkers of LUAD from the perspective of genes, so as to provide new thinking for gene-targeted therapy in LUAD.

GPCR (G protein-coupled receptor) is the largest cell membrane receptor. Previous studies have revealed that GPCR can regulate the cell migration, immune response, and metabolism [7]. As expected, GPCR is imbalanced in multiple cancers, and it also plays a crucial role in tumorigenesis and metastasis [8,9]. G protein-coupled receptor 37 (GPR37) is a member of GPCR, also known as parkin-associated endothelin receptor-like receptor [10], which has been studied in cancer as well. Wang et al. showed that GPR37 could be involved in promoting the metastasis of gastric cancer [11]. Conversely, GPR37 was poorly expressed in multiple myeloma cell adhesion model [12] and hepatocellular carcinoma [13]. Hence, one can see that GPR37 may be a double-edged sword in cancer, but its function in LUAD is rarely known.

In the present work, bioinformatics analysis revealed that transforming growth factor-β (TGF-β) pathway was associated with the expression of GPR37 in LUAD. TGF-β is a cytokine, which participates in numerous cell processes, especially the proliferation and metastasis of tumor cells [14]. Extensive studies have found that Smad protein can be phosphorylated by TGF-β signaling and participate in cell proliferation and apoptosis [15,16,17]. Recently, TGF-β/Smad signaling pathway has been found to function in tumor metastasis and progression, including metastasis of LUAD [18,19,20]. Since the mechanism of pathways regulation is very complicated, we selected this pathway for further study.

Herein, we aimed to assess whether GPR37 contributes to regulating the malignant phenotypes of LUAD cells, as well as the regulatory pathways. We first confirmed that GPR37 was expressed at high levels in LUAD and led to poor outcomes. Moreover, we revealed that GPR37 functioned as a potential regulator for the malignant progression of LUAD cells via regulating the TGF-β/Smad signaling pathway, which supplied a valuable breakthrough point for the therapy of LUAD.

## 2 Materials and methods

### 2.1 Bioinformatics analysis

The differential expression of GPR37 in LUAD patients was analyzed based on TCGA and Oncomine databases. The data downloaded from TCGA included 535 LUAD samples and 59 normal control samples. Also, we downloaded data from Oncomine database to further validate the GPR37 expression, including two LUAD datasets (Su Lung and Selamat Lung).

Kaplan–Meier survival analysis was exploited to assess the relevance between GPR37 expression and overall survival, and Cox regression analysis was utilized to analyze whether GPR37 can be used as an independent predictive factor of prognosis in LUAD patients. Moreover, GSEA analysis was performed to identify the pathways closely related to GPR37 expression.

### 2.2 Cell lines culture

Human LUAD cell lines A549, Calu-3, and LTEP-a-2 (American Type Culture Collection; ATCC, USA) as well as normal control cell BEAS2B (ATCC, USA) were routinely cultured in RPMI-1640 medium supplement with 10% FBS, 100 U/mL penicillin, and 0.1 mg/mL streptomycin at 37℃ with 5% CO_2_.

### 2.3 Cell transfection

Gain-of-function assays were performed with pcDNA3.1-GPR37, while si-GPR37#1 and si-GPR37#2 were used for loss-of-function assays. pcDNA3.1-GPR37, vector, si-GPR37#1 (5′-TTGGAAGCATTCACAAAGTA-3′), si-GPR37#2 (5′-CTTAATATCATCAGCCAGTT-3′), and si-con (5′-CGAACTCACTGGTCTGACC-3′) were all synthetized by Shanghai GenePharma Co., Ltd (Shanghai, China). Lipofectamine 2000 was utilized for all transfections in this work, on the basis of manufacturer’s standard. We identified 48 h as the harvest time of transfected cells.

### 2.4 RNA extraction and qRT-PCR

Trizol reagent (Invitrogen, Carlsbad, Calif) was utilized to extract total RNA from cells, and then the above RNA was reverse-transcribed into cDNA as directed by the standard of Reverse Transcription Kit (Takara, Dalian, China). qRT-PCR was conducted with SYBR (Toyobo, Japan) to detect the GPR37 quantitation, under the manufacturer’s standards. The data were computed by 2^−ΔΔCT^ method and normalized to GAPDH. The primers sequences were displayed as follows: GPR37: F: 5′-TTCTGCCTTCCGCTGGTCATCT-3′, R: 5′-TGAAGGTGGTGACTCCCAGAGA-3; GAPDH: F: 5′-TGTGTCCGTCGTGGATCTGA-3′, R: 5′-CCTGCTTCACCACCTTCTTGA-3′.

### 2.5 Western blot assay

Proteins were isolated from cells using Lysis buffer, and then 10% SDS–PAGE was exploited to detach the cells protein lysates. The proteins were transferred on to PVDF membrane, followed by blocking with Western Sealing Solution (5% skimmed milk powder) for 1 h. Then, the membranes were incubated with primary antibodies: anti-GPR37, anti-TGF-β1, anti-p-Smad2, anti-Smad2, anti-p-Smad3, anti-Smad3, and anti-GAPDH, and then with the secondary antibody (Santa Cruz Biotechnology, Inc., USA). All primary antibodies were obtained from Abcam (Cambridge, MA, USA). Finally, electrochemical luminescence (ECL, Thermo Fisher Scientific, Inc.) was utilized to develop the images.

### 2.6 Cell proliferation assays

CCK-8 and colony formation assays were conducted to assess the cell proliferation. For CCK-8 assay, cells were inoculated at the density of 1,000 per well in 96-well plates, and then cultured in a CO_2_ incubator at 37°C for 0, 24, 48, and 72 h. CCK-8 reagent was utilized to measure the cell viability, based on the manufacturer’s agreement. The OD value was detected by a microplate reader at 450 nm. For colony formation assay, trypsin was used to release cells from clusters and then counted cells. The cells were seeded in culture dish containing 5 mL preheated medium and cultured at 37°C with 5% CO_2_ for 1–2 weeks until the visible clone appeared. Afterwards, the cells were fixed in 4% paraformaldehyde and dyed with crystal violet.

### 2.7 Migration and invasion assays

Transwell chambers were used to assess the migratory and invasive capabilities of cells. In migration assay, cells were added to the upper chamber, and the complete culture solution as a chemoattractant was added to the matched lower chamber. After overnight, the cells without migrated ones were removed from the upper chamber, and the migrated cells were fixed with 4% polyformaldehyde and stained with crystal violet. Finally, cells were counted under the microscope. The invasion assay was similar to the migration assay, except for pre-coating matrix gel in Transwell room.

### 2.8 Statistical analysis

All assays were performed three times and data are presented as the mean ± SD. The statistical significance of parametric data was analyzed using student’s *t*-test (two groups) or one-way ANOVA variance analysis with post test of Dunnett (multiple groups). Kaplan–Meier survival analysis was utilized to assess the overall survival. The hazard ratio of variables in the univariable and multivariable modes was assessed using Cox Regression test. The difference has statistical significance at *P* < 0.05.

## 3 Results

### 3.1 GPR37 was overexpressed in LUAD tissues and cell lines and caused unoptimistic outcomes

**Figure 1:**
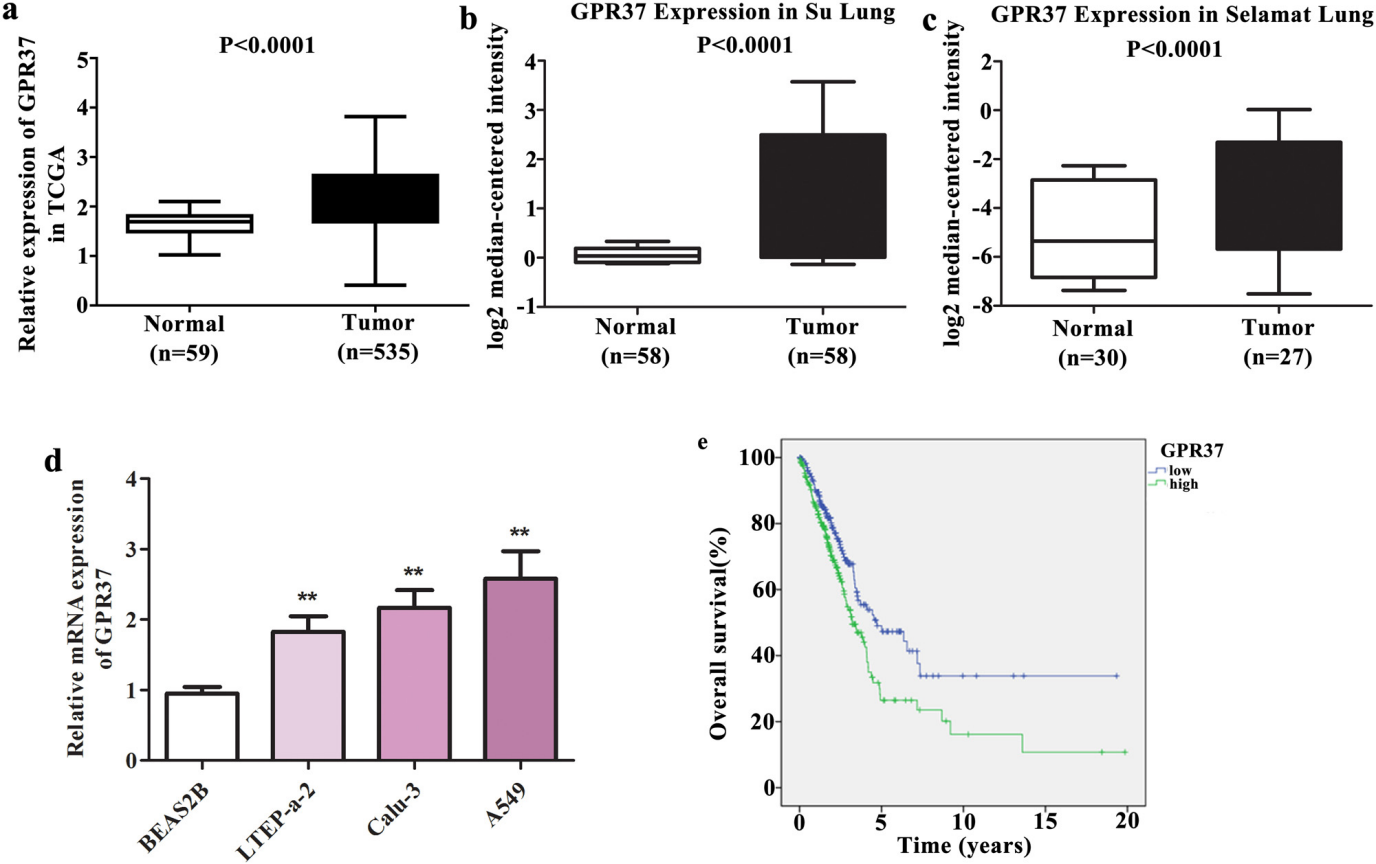
GPR37 was expressed at high levels in LUAD and was related to poor outcomes. The differential expression of GPR37 in LUAD patients and healthy control was analyzed based on the (a) TCGA database as well as Oncomine databases, (b) Su Lung, and (c) Selamat Lung. *P* < 0.0001. qRT-PCR was performed to measure (d) the expression of GPR37 in LUAD cell lines A549, Calu-3, and LTEP-a-2. **P < 0.01 vs control group. (e) Kaplan–Meier analysis was used to draw a conclusion between GPR37 expression and the survival rate in LUAD patients. *P* < 0.01.

To assess GPR37 expression in LUAD, we first download LUAD-associated data from TCGA and Oncomine databases. As presented in Figure 1a–c, GPR37 was obviously enhanced in LUAD tissues than that in normal control. Then, qRT-PCR was performed in LUAD cell lines (A549, Calu-3, and LTEP-a-2) and control cell (BEAS2B) to confirm this conclusion. The results revealed that the expression of GPR37 was strengthened in three LUAD cell lines compared with the control and expressed differently in different cells (Figure 1d). Here, A549 cells, which have the relatively higher expression, were selected for knockdown assays. Simultaneously, LTEP-a-2 cells were used for overexpression assays based on their relatively lower expression of GPR37.

In addition, Kaplan–Meier survival analysis indicated that high GPR37 expression indicated the shorter survival time in LUAD patients (Figure 1e). As presented in Table 1 and Supplementary Table S1, we selected 474 cases with complete clinical data (including age, gender, stage, T stage, M, and N) for prognostic analysis and Cox regression analysis. Notably, we found that GPR37 could be used as an independent predictive factor of prognosis in patients with LUAD, according to the results from Cox regression analysis (Table 2). These data prompted that GPR37 may play a major role in the malignant progression of LUAD.

**Table 1:** Clinical data of LUAD patients in this work

Characteristics	Total, *n* (%) *n* = 474
**Age**	
<60	129 (27.2%)
≥60	345 (72.8%)
**Gender**	
Female	256 (54.0%)
Male	218 (46.0%)
**Stage**	
I + II	375 (79.1%)
III + IV	99 (20.9%)
**Pathologic-T**	
T1 + T2	413 (87.1%)
T3 + T4	61 (12.9%)
**Pathologic-N**	
N0	315 (66.5%)
N1 + N2 + N3	159 (33.5%)
**Pathologic-M**	
M0	453 (95.6%)
M1	21 (4.4%)

**Table 2:** Cox regression analysis indicated that GPR37 is an independent predictor of lung adenocarcinoma prognosis

Variables	Univariate analysis			Multivariate analysis		
	*P* value	HR	95% CI	*P* value	HR	95% CI
GPR37 expression (high/low)	0.003*	1.570	1.160–2.124	0.001*	1.668	1.223–2.275
Clinical-Stage (I + II/III + IV)	0.000*	2.388	1.737–3.282	0.484	1.173	0.750–1.833
Pathologic-T (T1 + T2/T3 + T4)	0.000*	2.228	1.519–3.266	0.001*	2.035	1.322–3.132
Pathologic-M (M0/M1)	0.021*	1.948	1.105–3.433	0.209	1.497	0.798–2.810
Pathologic-N (N0/N1 + N2 + N3)	0.000*	2.483	1.841–3.348	0.000*	2.095	1.462–3.001
Age (<60/≥60)	0.752	1.056	0.753–1.480			
Gender (female/male)	0.520	1.103	0.819–1.486			

HR: hazard ratio. * Indicated the difference is significant.

### 3.2 Cell transfection efficiency

**Figure 2:**
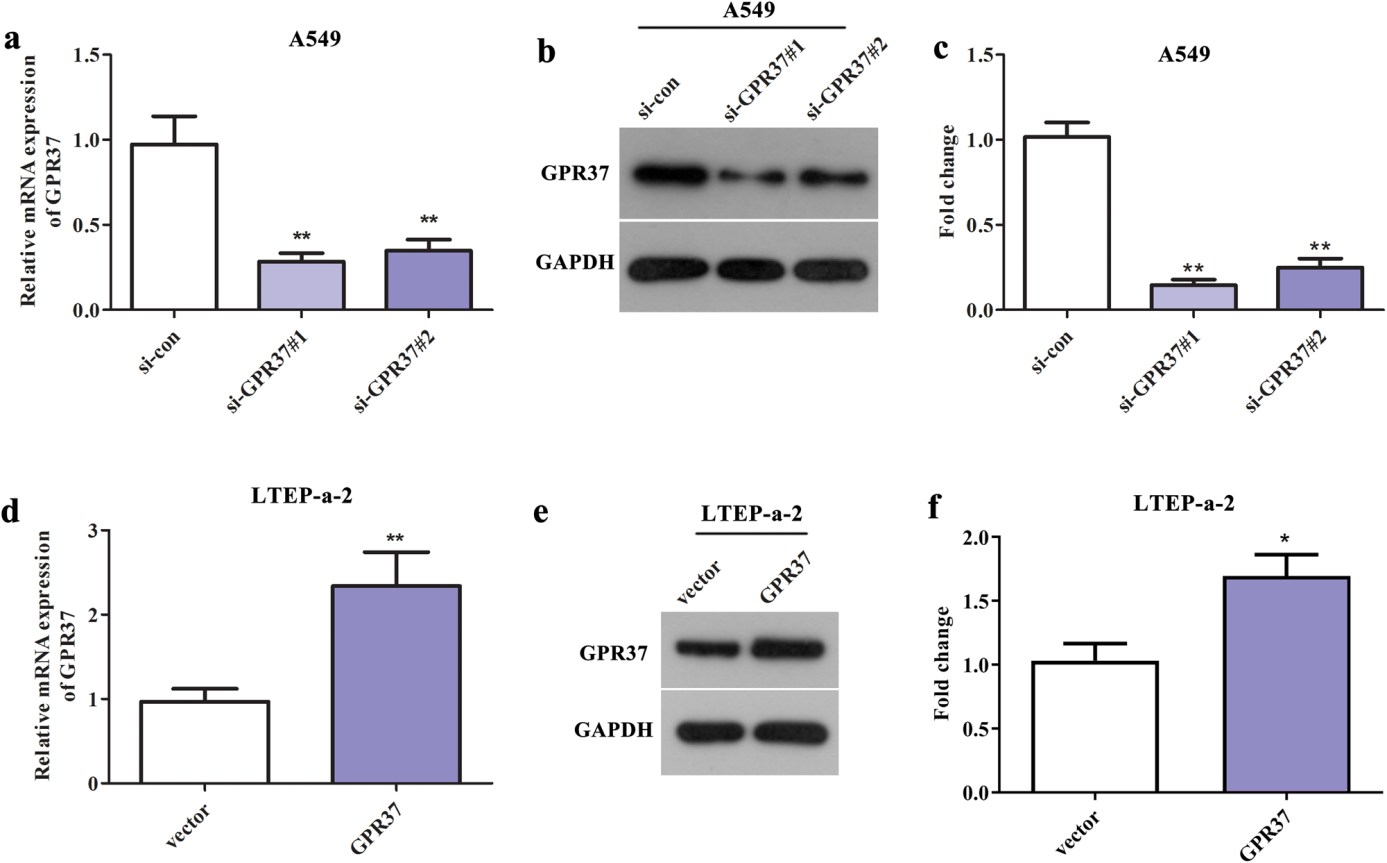
The transfection efficiency of GPR37 downregulation/upregulation in LUAD cell lines A549 and LTEP-a-2. qRT-PCR and western blot assays were performed to measure the (a) mRNA and (b and c) protein levels of GPR37 in A549 cells after transfected with si-GPR37#1 and si-GPR37#2. **P < 0.01 vs si-con group. Concurrently, the (d) mRNA and (e and f) protein levels of GPR37 in LTEP-a-2 cells after transfected with pcDNA3.1-GPR37 were also detected using qRT-PCR and western blot assay. **P < 0.01 vs vector group. *P < 0.05 vs vector group.

In order to explore the function of GPR37 in LUAD, we first transfected A549 cells with si-GPR37#1, si-GPR37#2, and si-con and transfected LTEP-a-2 cells with pcDNA3.1-GPR37 and vector. Then, qRT-PCR and western blot assay were performed to measure the GPR37 expression in transfected cells. As displayed in Figure 2a–c, GPR37 expression was obviously declined following transfection with si-GPR37#1 and si-GPR37#2. Of note, si-GPR37#1 has a relatively higher knockdown efficiency; therefore, we opted for si-GPR37#1 for subsequent loss-of-function assays. Consistently, after LTEP-a-2 cells were transfected with pcDNA3.1-GPR37, the mRNA and protein levels of GPR37 were markedly escalated than that in vector group (Figure 2d–f). These results indicated that the transfections were successful in both A549 and LTEP cells.

**Figure 3:**
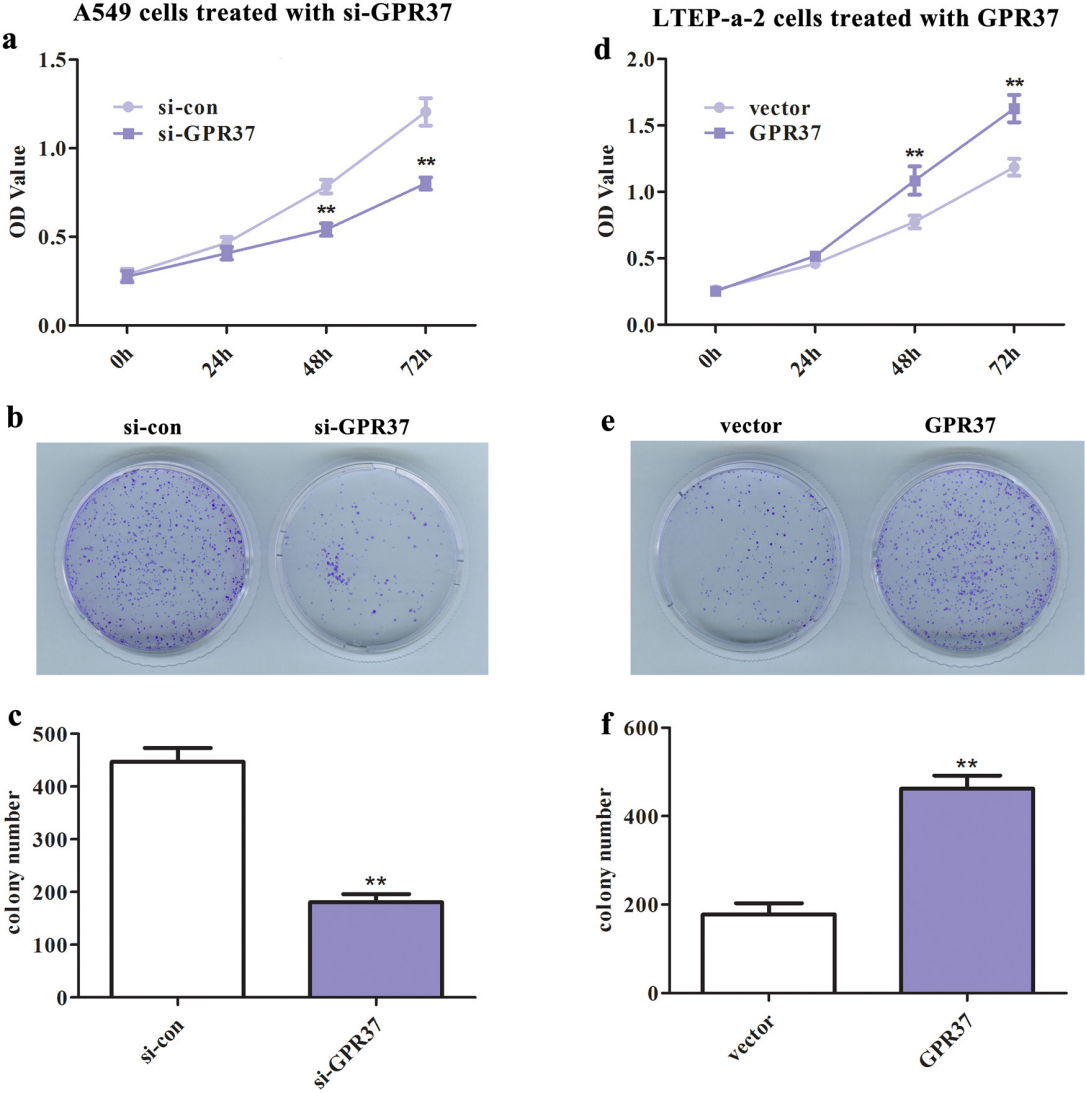
GPR37 contributed to promoting the proliferation of LUAD cells. CCK-8 assay was carried out to measure the viability of (a) A549 cells treated with si-GPR37 (**P < 0.01 vs si-con group) and (d) LTEP-a-2 cells treated with pcDNA3.1-GPR37 (**P < 0.01 vs vector group). Colony formation assay was conducted to assess the proliferative capacity of (b and c) A549 cells treated with si-GPR37 (**P < 0.01 vs si-con group) and (e and f) LTEP-a-2 cells treated with pcDNA3.1-GPR37 (**P < 0.01 vs vector group).

**Figure 4:**
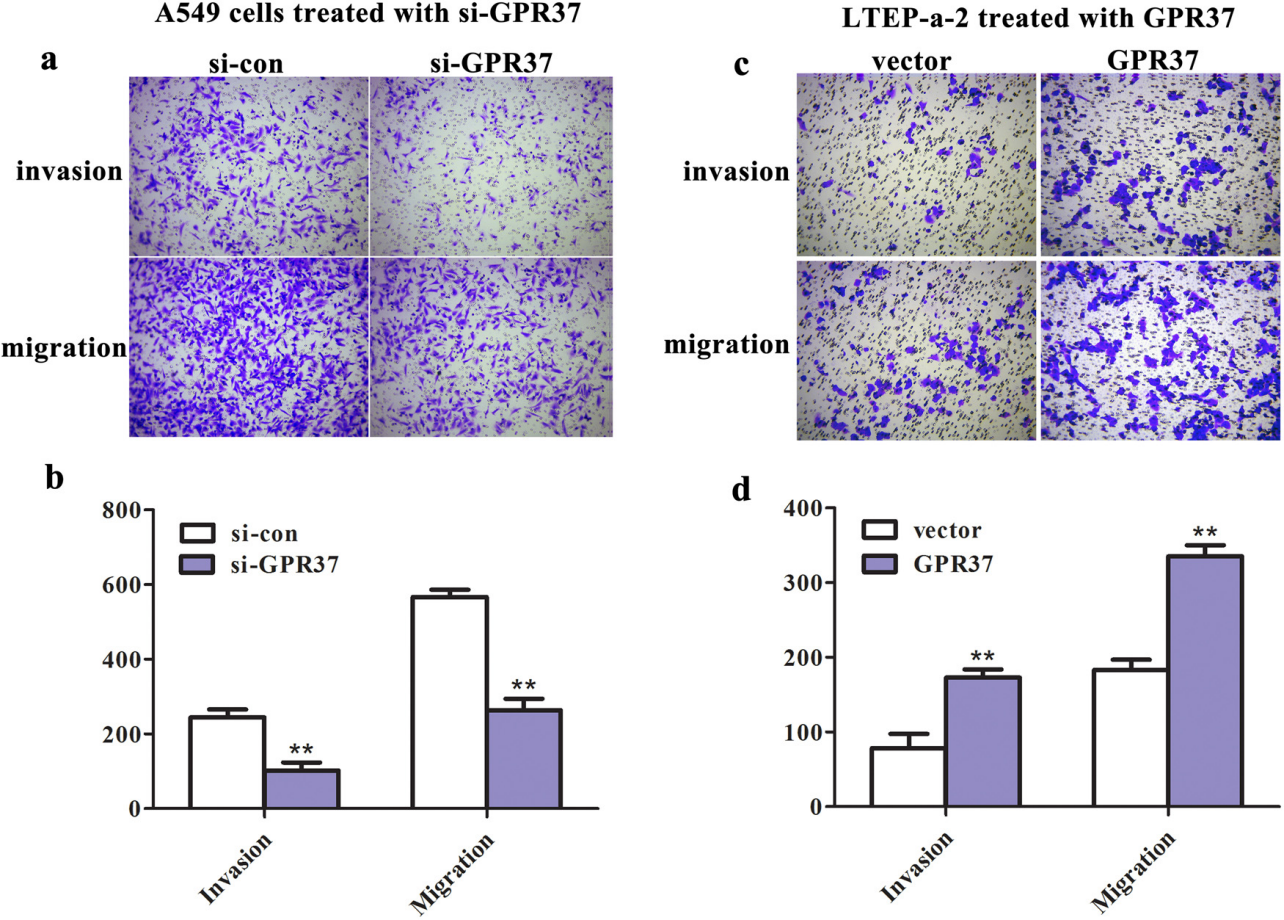
GPR37 contributed to promoting the invasion and migration of LUAD cells. Transwell assay was carried out to measure the invasive and migratory abilities of (a and b) A549 cells treated with si-GPR37 (**P < 0.01 vs si-con group) and (c and d) LTEP-a-2 cells treated with pcDNA3.1-GPR37 (**P < 0.01 vs vector group).

GPR37 contributed to promoting the malignant biological behaviors in LUAD cells *in vitro.* With results understanding the upregulation of GPR37 in LUAD, the next focus was diverted to the impact of GPR37 on the phenotype of LUAD cells. The cell proliferative capacity was determined using CCK-8 and colony formation assays. As presented in Figure 3a–c, the viability and colony number were markedly declined following the transfection with si-GPR37 in A549 cells, whereas the proliferative ability showed a remarkable increase after overexpression of GPR37 in LTEP-a-2 cells (Figure 3d–f). Similarly, Transwell assay was carried out to measure the invasion and migration of LUAD cells. As expected, the number of invading and migrating cells was markedly decreased in GPR37-lacked cells (Figure 4a and b), while overexpression of GPR37 in LTEP-a-2 cells resulted in a noticeable promotion in the invasive and migratory abilities of cells (Figure 4c and d). All these data demonstrated that GPR37 plays a major role in the phenotype regulation of LUAD cells.

### 3.3 Silence of GPR37 hampers the activation of TGF-β/Smad pathway in LUAD cells

**Figure 5:**
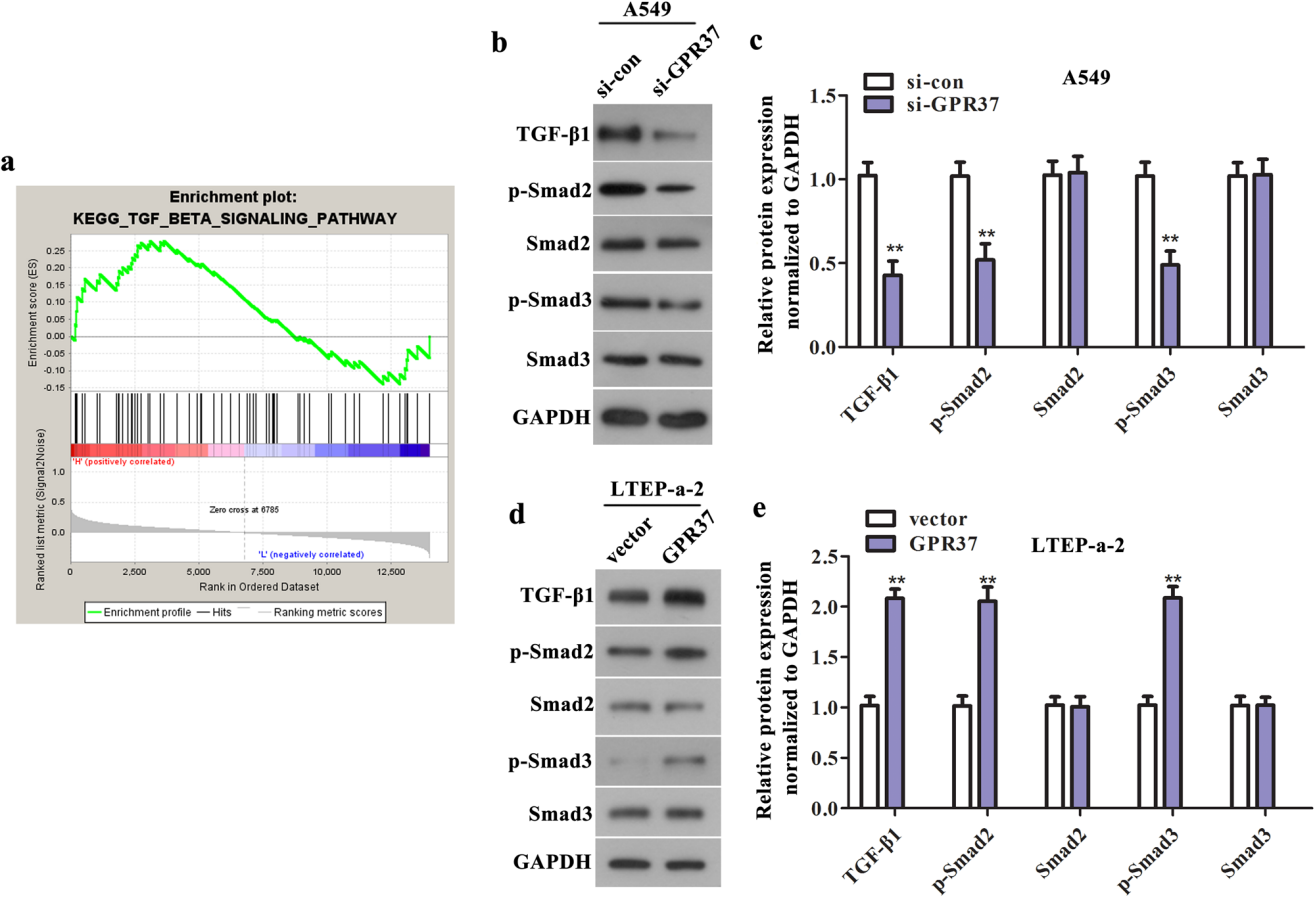
TGF-β/Smad pathway mediated the modulation of GPR37 on the malignant phenotype of LUAD cells *in vitro.* (a) GSEA analysis was performed to identify the pathways closely related to GPR37 expression. Then, western blot assay was performed to measure the expression levels of TGF-β/Smad pathway-related factors in (b and c) A549 cells treated with si-GPR37 (**P < 0.01 vs si-con group) and (d and e) LTEP-a-2 cells treated with pcDNA3.1-GPR37 (**P < 0.01 vs vector group).

To probe how GPR37 functioned in LUAD cells, GSEA analysis was performed to identify the pathways closely related to GPR37 expression. Five hundred and thirty five LUAD samples were grouped according to the high and low expression of GPR37. We selected the top 25% (134 samples) with the highest GPR37 expression level and the last 25% (133 samples) with the lowest GPR37 expression level for KEGG pathway enrichment difference analysis. The specific sample information is shown in heat_ map (Supplementary Figure S1). As a result, TGF-β pathway was associated with the expression of GPR37 (Figure 5a). Western blot assay was used to measure the expression of TGF-β pathway-related proteins, which indicated that downregulation of GPR37 caused a decrease of TGF-β1 as well as the extents of Smad2 and Smad3 phosphorylation. Nevertheless, the expression of total Smad2 and Smad3 was almost unchanged (Figure 5b and c). Also, as displayed in Figure 5d and e, TGF-β1 expression and the phosphorylation of Smad2 and Smad3 were clearly enhanced after LTEP-a-2 cells transfected with pcDNA3.1-GPR37. These findings demonstrated that TGF-β/Smad pathway mediated the modulation of GPR37 on the malignant phenotype of LUAD cells *in vitro.*

## 4 Discussion

In this work, we indicated that GPR37 was expressed at high levels in LUAD tissues and cell lines. Higher expression of GPR37 foreboded a shorter survival time, and GPR37 could be used as an independent predictive factor of prognosis in patients with LUAD. Additionally, upregulation of GPR37 contributed to promoting the proliferation, migration, and invasion of LUAD cells *in vitro,* which was realized at least partially through TGF-β/Smad singling pathway.

The development of tumor cells cannot be separated from the surrounding tumor microenvironment, and the information exchange between cells and the outside is closely related to GPCR. Yan et al. showed that GPR87 enhanced the metastasis of CD133^+^ stem cells in hepatocellular carcinoma cells [21]. GPR56, as an adhesion receptor, is highly expressed in many tumor cells, such as gliomas [22], melanoma [23], and acute lymphoblastic leukemia [24], and participates in the occurrence and development of tumors. In hepatocellular carcinoma, patients with low GPR37 expression had a shorter survival time, which may be due to the inhibition of proliferation of HCC cells caused by downregulation of GPR37 [13]. Huang et al. found that GPR37 was expressed at high levels in proliferative multiple myeloma cells [12]. In a recent study, Wang et al. found that GPR37 was upregulated in LUAD patients with TP53/EGFR co-mutation [25], while TP53/EGFR co-mutation made LUAD patients resistant to chemotherapy, resulting in poor prognosis [26]. Our data revealed a notable enhancement in the expression of GPR37 in LUAD, which was associated with poor outcomes. Moreover, GPR37 could be used as an independent predictive factor of prognosis in patients with LUAD. The data of functional experiment demonstrated that suppression of GPR37 results in an apparent decrease in the proliferation, migration, and invasion of LUAD cells, prompting that GPR37 may be a novel biomarker of malignant progression of LUAD.

With more evidences showing that TGF-β plays an important role in the development of tumor, inhibiting TGF-β signaling has become a new thinking in cancer treatment [27]. Generous studies indicated that TGF-β signaling pathway has dual activities of tumor suppressor and promoter [28,29]. In the early stage of cancer, TGF-β signaling pathway can inhibit the proliferation of cancer cells, but with the development of cancer, it can in turn promote the proliferation and invasive ability of cancer cells [30,31,32]. Smad protein is the main specific intracellular signal transducer in TGF-β1 signal transduction pathway, and smad2,3 play an important role in TGF-β signal transduction [33]. Furthermore, extensive studies have shown that TGF-β signaling pathway is involved in the malignant progression of LUAD [20,34,35]. Importantly, Marini KD et al. found that TGF-β signaling is often activated in advanced LUAD and is related to poor prognosis [36]. In this paper, upregulation of GPR37 can obviously enhance the expression of TGF-β1 as well as the extents of Smad2 and Smad3 phosphorylation, thus activating the TGF-β1/Smad pathway, while knockdown of GPR37 resulted in an opposite outcome. From all the above, we revealed that the regulation of GPR37 on the malignant progression of LUAD cells may be achieved via TGF-β/Smad pathway *in vitro*. However, the exact mechanism is not very clear and in-depth research is needed.

To sum up, our findings indicated that GPR37 was expressed at high levels in LUAD and related to poor outcomes. The promoting effect of GPR37 on malignant biological behaviors was realized via TGF-β/Smad pathway in LUAD cells *in vitro*. This work provided a certain experimental basis for GPR37-targeted therapy in LUAD, but whether the *in vitro* and *in vivo* experiments are consistent has not been determined, and it is still to be further studied to fully reveal its function in the development of LUAD.
